# Higher efficacy of intravenous thrombolysis in patients with acute ischemic stroke taking direct oral anticoagulants—A new relevant hypothesis

**DOI:** 10.3389/fneur.2024.1458697

**Published:** 2024-09-06

**Authors:** Senta Frol, Janja Pretnar Oblak, Pawel Kermer, George Ntaios, Panagiotis Papanagiotou, Mišo Šabovič

**Affiliations:** ^1^Department of Vascular Neurology, University Medical Center Ljubljana, Ljubljana, Slovenia; ^2^Faculty of Medicine, University of Ljubljana, Ljubljana, Slovenia; ^3^Department of Neurology, Nordwest-Krankenhaus Sanderbusch, Friesland Kliniken GmbH, Sande, Germany; ^4^University Medical Center Göttingen, Göttingen, Germany; ^5^Department of Internal Medicine, Faculty of Medicine, School of Health Sciences, University of Thessaly, Larissa, Greece; ^6^Clinic of Diagnostic and Interventional Neuroradiology, Klinikum Bremen Mitte, Bremen, Germany; ^7^Department of Radiology, Aretaieion University Hospital, National and Kapodistrian University of Athens, Athens, Greece; ^8^Department of Vascular Disorders, University Medical Center Ljubljana, Ljubljana, Slovenia

**Keywords:** direct oral anticoagulants, intravenous thrombolysis, functional outcome, enhanced fibrinolytic activity, thrombi lysability

## Introduction

Direct oral anticoagulants (DOACs) have been established as first-line therapy for stroke prevention in patients with non-valvular atrial fibrillation due to their high safety and efficacy, as demonstrated in large randomized controlled trials (RCTs) (1–4) and real-world data. Despite their efficacy, about 1%−2% of DOAC-treated patients suffer from acute ischemic stroke (AIS) (1–4). At the same time, intravenous thrombolysis (IVT) is recommended as first-line therapy for AIS patients (5, 6). Currently, alteplase is the preferred thrombolytic agent, while tenecteplase, which is more fibrin-specific and has a longer half-life, has recently been approved for IVT in AIS in Europe (7). However, most international guidelines advise against IVT in DOAC-treated patients who have ingested their medication within 48 h prior to AIS onset, except for dabigatran-treated patients reversed by idarucizumab (5, 8).

Ongoing debates regarding IVT safety in patients on DOACs speculate on possible pathophysiological explanations. It was hypothesized that both direct and indirect thrombin inhibition might reduce disruptions to the blood-brain barrier, thereby lowering the risk of hemorrhage (9, 10). Equally important is the high efficacy of IVT in DOAC-treated patients. Recently, no safety concerns regarding IVT were reported while patients receiving IVT were more likely to have good functional outcomes (11, 12).

In this context, this opinion article discusses about the potentially higher efficacy of IVT in patients on DOACs, a topic which warrants more in-depth exploration, such as enhanced fibrinolytic activity.

## Clinical studies

Multiple studies including observational studies (10, 12), reviews and meta-analysis (13, 14) revealed safety of IVT with no increased risk of symptomatic intracranial hemorrhage in patients with recent DOAC intake.

Through a literature search, we identified eight articles addressing IVT efficacy in DOAC-treated patients (10–12, 15–19). Higher IVT efficacy was reported in two most recent studies (11, 12), reporting better functional outcomes in DOAC-treated patients. Eighty-one percent of IVT-treated patients achieved an mRS score of 0–2 at 90 days compared to 28% of non-IVT-treated patients (11) and DOAC-treated patients had significantly better functional outcome at discharge (12). A meta-analysis of 6 observational studies conducted by Behnoush et al. found that DOAC-treated patients undergoing IVT were at higher risk of functional dependence following thrombolysis than patients not receiving anticoagulation therapy (15). Due to a lack of data, the results were not adjusted for patient comorbidities and stroke severity. In contrast, Kam et al. (12) showed in an adjusted analysis that DOAC-treated patients have better neurological outcomes after accounting for various baseline characteristics. As all the studies are retrospective, the selection bias could influence the results, which is why further prospective studies are preferable.

Further studies are expected in the near future. An update of the retrospective data from the DOAC International Thrombolysis Trial & Registry will be available soon, and the prospective study is currently collecting data to further investigate this important clinical issue.

## Theoretical background

### Improved lysability of thrombi

A hypothesis of possible higher sensitivity of thrombi in DOACs treated patients to IVT was previously suggested by several members of our group (20). In the clinical setting, the lysability of stroke thrombi strongly depends on thrombus structure (21). Thrombi have different structural compositions: fibrin- and platelet-rich areas feature a dense, compacted network of thin fibers with entrapped platelets, while red blood cell-rich areas have a loose, poorly compacted network of thick fibrin fibers with red blood cells (22). Stroke thrombi are typically heterogeneous and contain fibrin, platelets, red blood cells, von Willebrand factor, and neutrophil extracellular traps. When retrived stroke thrombi were analyzed, it was found that red blood cell-rich thrombi were much more susceptible to lysis by t-PA than fibrin-rich thrombi (23). As current imaging techniques do not allow adequate visualization of the composition of stroke thrombi *in vivo*, histological analysis of retirived stroke thrombi remain the basic method to explore the relationship between thrombus composition and its susceptibility to lysis. Histological studies suggest that cardioembolic thrombi are predominantly composed of fibrin- and platelet-rich parts, whereas noncardioembolic thrombi are mainly associated with red blood cell-rich parts (24). Consequently, cardioembolic thrombi may be less susceptible to IVT with t-PA. Red blood cell-rich thrombi are associated with a fibrin structure and a looser thrombus architecture, which leads to an enlargement of the pores within the fibrin networks, allowing increased penetration of t-PA into the thrombi and consequently a better outcome of fibrinolysis (25, 26). It is important to note that thrombin plays an important role in modulating thrombus structure: high thrombin concentrations lead to the formation of a dense network of thin fibers, resulting in fibrin-rich thrombi with entrapped platelets. In contrast, low thrombin concentrations produce a loose, porous fibrin network with thick fibrin fibers, facilitating the formation of red blood cell-rich thrombi (27, 28). Thin fibrin fibers are less susceptible to t-PA-induced thrombolysis than thick fibrin fibers (29). Trapped platelets in fibrin-rich thrombi could reduce the efficacy of thrombolysis by releasing fibrinolysis inhibitors such as anti-2-antiplasmin and plasminogen activator inhibitor (PAI-1) and factor XIII, which cross-link the fibrin network, and by thrombus compaction through platelet-induced thrombus retraction (30). Overall, fibrin structure appears to be an important determinant of thrombus composition and susceptibility to thrombolysis, so agents that affect fibrin structure could influence the efficacy of thrombolysis.

Dabigatran, a thrombin inhibitor, appears to affect the structure of stroke thrombi and their susceptibility to IVT by lowering thrombin concentration (31). Factor Xa inhibitors work by directly inhibiting the activity of factor Xa, a crucial enzyme in the coagulation cascade responsible for the conversion of prothrombin to thrombin. This inhibition reduces thrombin and, consequently, the formation of fibrin which is the main component of thrombi (32, 33). This issue and its clinical relevance need to be further investigated.

Under laboratory conditions, plasma clots formed in the presence of dabigatran exhibit an altered structure that is more susceptible to IVT (28). These clots have a looser, less rigid, and more permeable fibrin network with thicker fibers. Additionally, dabigatran reduces the activity of activated thrombin-activatable fibrinolysis inhibitor (TAFI). Both TAFI-dependent and TAFI-independent mechanisms could increase the susceptibility of thrombi formed in the presence of dabigatran to IVT with t-PA (28). Similar laboratory results were obtained with rivaroxaban. Varin et al. (34) have shown that pretreatment with rivaroxaban increases the lysability of whole blood and plasma clots *in vitro*. They showed that rivaroxaban reduces thrombin generation due to platelets and red blood cells, which leads to a change in the fibrin network characterized by a looser plasma fibrin network with thicker fibers and larger pores. As a result, the permeation of fibrinolytic agents into the clot is increased, making the clot more susceptible to thrombolysis. This profibrinolytic potential of rivaroxaban was further enhanced by the inhibition of TAFI activation (34).

### Influence of DOACs on mechanical thrombectomy

Mechanical thrombectomy (MT) is a crucial reperfusion treatment in patients with AIS (35). The use of DOACs raises questions about their potential influence on the success of MT. Previous meta-analyses have indicated that MT is safe for patients on anticoagulation therapy. The composition of thrombi, affected by anticoagulants, plays a critical role in MT success (36). Erythrocyte-rich thrombi are easier to retrieve mechanically than fibrin-rich thrombi (36). By altering thrombus composition, DOACs may enhance MT efficacy, potentially making thrombi easier to remove (36, 37). However, studies have shown that clots from patients on anticoagulation therapy tend to be stiffer and more challenging to fully recanalize (38). Despite these differences, a previous meta-analysis found no significant difference in successful recanalization rates between anticoagulated and non-anticoagulated patients (39). Therefore, while there is a hypothesis that DOACs might facilitate easier thrombectomy, further investigation is warranted to fully understand their influence on thrombus composition and recanalization outcomes.

## Conclusion and future perspective

Current findings generate the hypothesis that DOAC therapy has benefical effects not only on the ischemic stroke prevention but also on the more efficient and safer revascularization therapy by IVT and possibly also MT. Prospective studies of appropriate size and systematic collection of data from daily clinical practice regarding higher efficacy of recanalization therapies in patients with AIS taking DOACs are needed to clarify this issue and its clinical significance. Given the fact that IVT is increasingly being used in AIS patients receiving DOACs, a large amount of data is expected that would enable appropriate studies. Of course, RCTs would be the best approach to test the hypothesis, but they are not feasible.
